# Fungal metabolic gene clusters—caravans traveling across genomes and environments

**DOI:** 10.3389/fmicb.2015.00161

**Published:** 2015-03-03

**Authors:** Jennifer H. Wisecaver, Antonis Rokas

**Affiliations:** Department of Biological Sciences, Vanderbilt UniversityNashville, TN, USA

**Keywords:** metabolic gene cluster, gene innovation, horizontal gene transfer, microbial ecology, comparative genomics, secondary metabolism, specialized metabolism, physical linkage

## Abstract

Metabolic gene clusters (MGCs), physically co-localized genes participating in the same metabolic pathway, are signature features of fungal genomes. MGCs are most often observed in specialized metabolism, having evolved in individual fungal lineages in response to specific ecological needs, such as the utilization of uncommon nutrients (e.g., galactose and allantoin) or the production of secondary metabolic antimicrobial compounds and virulence factors (e.g., aflatoxin and melanin). A flurry of recent studies has shown that several MGCs, whose functions are often associated with fungal virulence as well as with the evolutionary arms race between fungi and their competitors, have experienced horizontal gene transfer (HGT). In this review, after briefly introducing HGT as a source of gene innovation, we examine the evidence for HGT's involvement on the evolution of MGCs and, more generally of fungal metabolism, enumerate the molecular mechanisms that mediate such transfers and the ecological circumstances that favor them, as well as discuss the types of evidence required for inferring the presence of HGT in MGCs. The currently available examples indicate that transfers of entire MGCs have taken place between closely related fungal species as well as distant ones and that they sometimes involve large chromosomal segments. These results suggest that the HGT-mediated acquisition of novel metabolism is an ongoing and successful ecological strategy for many fungal species.

## Genetic nomads—gene innovation through horizontal gene transfer

The ability to respond to dynamic ecological pressures, such as shifts in nutrient availability or biological interactions, is a defining characteristic of many successful species and often requires evolutionary innovation. Horizontal gene transfer or HGT—the transfer of genetic material from one organism to another through a process other than reproduction—is one source of innovation that can result in the rapid acquisition of genes that contribute to ecologically important traits (Gogarten and Townsend, 2005). In bacteria and archaea, HGT is a major contributor to gene innovation (Ochman et al., 2000; Boucher et al., 2003; Jain et al., 2003; Treangen and Rocha, 2011), with as much as 32% of genes in these organisms, depending on the species, having been recently acquired via HGT (Koonin et al., 2001), and with over 75% of prokaryotic genes having experienced at least one HGT event (Dagan et al., 2008; Kloesges et al., 2011).

Although once considered a process of limited effect outside of prokaryotes, we now know that HGT has occurred in all major eukaryotic lineages (reviewed by Huang, 2013), including multicellular plants (e.g., Yue et al., 2012; Li et al., 2014) and even animals, whose isolated germlines were once considered effectively inaccessible to foreign DNA (e.g., Dunning Hotopp et al., 2007; Graham et al., 2008; Danchin et al., 2010; Moran and Jarvik, 2010; Boschetti et al., 2011). Although the frequency of HGT is generally substantially lower in eukaryotic genomes compared to prokaryotic ones (Keeling and Palmer, 2008; Andersson, 2009), notable examples that invoke as well as showcase HGT's influence on eukaryotic evolution include the green plant radiation onto dry land (Yue et al., 2012), the repeated colonization of animal digestive tracts by microbial eukaryotes (Garcia-Vallve et al., 2000; Ricard et al., 2006), and even adaptation to life in boiling acid lakes in extremophile algae (Schönknecht et al., 2013). Elevated rates of HGT have also been coincident with the loss of typical eukaryotic traits such as sexual reproduction (Boschetti et al., 2012) and aerobic growth (Andersson et al., 2003; Loftus et al., 2005; Pombert et al., 2012).

Among eukaryotic lineages, the fungi are no exception and also show HGT-driven gene innovation, broadly resulting in expanded repertoires of secreted and transporter proteins and increased metabolic capacities (Richards et al., 2011). A survey of sixty fungal genomes detected hundreds of genes horizontally acquired from bacteria (Marcet-Houben and Gabaldon, 2010), and studies suggest that bacteria-derived genes serve various functions in diverse fungal lineages, from vitamin biosynthesis in yeast (Hall and Dietrich, 2007), to the production of secondary metabolites in filamentous fungi (van den Berg et al., 2008; Schmitt and Lumbsch, 2009; Lawrence et al., 2011). Other cases of HGT from bacteria have been implicated in the ability of some soil-dwelling fungi to utilize unusual carbon sources (Wenzl et al., 2005), host adaptation in fungal pathogens (Hu et al., 2014), and adaptation to anoxic environments in the model fermenter *Saccharomyces cerevisiae* (Gojkovic et al., 2004; Hall et al., 2005). Aside from bacteria, the list of donors of fungal genetic material that was acquired via HGT includes plants (Richards et al., 2009), microbial eukaryotes (Slot and Hibbett, 2007; Tiburcio et al., 2010), and, perhaps most frequently, other fungi (Wisecaver et al., 2014).

## Molecular tariffs—pathway complexity as a barrier to HGT

Phylogenomic surveys suggest that HGT has affected between 0.1–2.8% of genes on a typical fungal genome (Marcet-Houben and Gabaldon, 2010; Wisecaver et al., 2014). It may seem surprising that HGT in fungi is not more common, given the obvious advantage of acquiring a pre-adapted gene from the environment rather than *de novo* from non-coding sequence or through gene duplication and subsequent functional specialization. However, the structure of the eukaryotic cell, in which the genome is tightly packaged with chromatin and compartmentalized in the nucleus, as well as incompatibilities between potential donor and recipient organisms in their genome architecture or molecular machinery (e.g., differences in promoter regions, intron-splicing, and codon usage patterns), are all likely barriers to rampant HGT in eukaryotes (Keeling and Palmer, 2008; Richards et al., 2011). Fungal biology may also limit the opportunity of DNA exchange; many species of filamentous fungi grow through branching and fusion of hyphal mycelium and have evolved vegetative incompatibility mechanisms to limit cell fusion with genetically different individuals (Glass et al., 2000).

Studies of HGT in prokaryotes suggest that the propensity of a gene to undergo HGT is strongly associated with its biological function. For example, genes involved in replication, translation and transcription are transferred less often than genes participating in cellular metabolism (Jain et al., 1999; Nakamura et al., 2004). Cohen et al. (2011) argued that this trend is likely driven by the correlated factor of gene connectivity; genes whose products form many complex interactions are less likely to undergo successful HGT because they are less likely to be successfully integrated into a foreign system. In contrast, genes whose products function alone, without interacting with other proteins, are predicted to be the most amenable to HGT (Moran et al., 2012). Thus, the fact that eukaryotes have large genomes with complex gene interaction networks (Szklarczyk et al., 2015) may further explain partly why HGT is not as abundant in these organisms compared to prokaryotes.

## Metabolic caravans—gene clustering can facilitate the transfer of entire pathways

The majority of high-throughput analyses in fungi and other organisms has examined and quantified the impact of HGT on a gene-by-gene basis, effectively assuming that each gene that has undergone HGT has done so independently of any other genes (e.g., Loftus et al., 2005; Dagan et al., 2008; Marcet-Houben and Gabaldon, 2010; Cohen et al., 2011; Kloesges et al., 2011; Boschetti et al., 2012; Yue et al., 2012; Schönknecht et al., 2013; Wisecaver et al., 2014). Yet, numerous discoveries in diverse fungal organisms have shown that multiple genes can be transferred together (Slot and Hibbett, 2007; Khaldi et al., 2008; Novo et al., 2009; Moran and Jarvik, 2010; Slot and Rokas, 2010, 2011; Khaldi and Wolfe, 2011; Campbell et al., 2012; Cheeseman et al., 2014; Greene et al., 2014). For example, the genome of a *S. cerevisiae* commercial wine strain contains a 17-kb DNA segment (including five protein coding genes with various functions) that was horizontally acquired from the yeast *Zygosaccharomyces bailii*, a common wine fermentation contaminant (Novo et al., 2009). More often, however, co-transferred genes are involved in the same function, effectively reducing the connectivity barrier to HGT. For example, two genes required for carotenoid biosynthesis in pea aphids were horizontally transferred in a single event from fungi and are responsible for a red-green color polymorphism in these insects that influences their susceptibility to predators (Moran and Jarvik, 2010).

An important prerequisite for the co-transfer of functionally associated genes is the genes' physical proximity in the donor genome. Although it was once assumed that the order of the genes in eukaryotic genomes was random, limiting opportunity for such co-transfers, a slew of studies suggests that this is not true (Lee, 2003; Hurst et al., 2004). In fungi, this non-randomness is perhaps best illustrated by metabolic gene clusters (MGCs), which typically consist of metabolic pathways whose constituent genes are physically linked in the genome. MGCs are common features of fungal genomes (Keller and Hohn, 1997; Hall and Dietrich, 2007; Wisecaver et al., 2014), and similar MGCs have also been discovered in several plant species (e.g., Frey et al., 1997; Qi et al., 2004; Field and Osbourn, 2008; Winzer et al., 2012; Itkin et al., 2013).

In fungi, MGCs most often code for metabolic pathways that are not required to sustain cellular life but instead confer accessory traits that allow organisms to better respond to ecological pressures. Fungal examples of such MGCs include those for resistance to xenobiotic arsenic (Bobrowicz et al., 1997) and degradation of plant defensive compounds (Greene et al., 2014) as well as utilization of uncommon or ecologically specialized forms of carbon (Hittinger et al., 2004), nitrogen (Jargeat et al., 2003; Wong and Wolfe, 2005) and other nutrients (Hull et al., 1989; Hall and Dietrich, 2007). Perhaps the most well-known fungal MGCs are those that code for the production of secondary metabolites (SMs) (Keller and Hohn, 1997; Keller et al., 2005)—the focus of this special Research Topic.

A growing number of studies shows that several fungal MGCs encoding diverse specialized metabolic pathways have undergone HGT (Table 1). Some of the transferred MGCs are involved in nutrient acquisition, including the *GAL* cluster for galactose utilization (Slot and Rokas, 2010) and the fHANT-AC cluster for nitrate assimilation (Slot and Hibbett, 2007), whereas others are found in plant-associated filamentous fungi and appear to have been acquired for degrading defensive SMs produced by plant hosts (Greene et al., 2014). However, most transferred MGCs are involved in the production of fungal SMs, including sterigmatocystin (Slot and Rokas, 2011), bikaverin (Campbell et al., 2012), fumonisin (Khaldi and Wolfe, 2011), and gliotoxin (Patron et al., 2007).

**Table 1:** **Summary of published cases of HGT involving MGCs**.

**MGC**	**Donor^a^**	**Recipient^a^**	**No. genes^b^**				**Function**	**References**
			**D**	**R**	**T**	**C**		
ACE1 biosynthesis	*Magnaporthe*	*Aspergillus*	15	6	5	6	SM production	Khaldi et al., 2008^c^
Bikaverin biosynthesis	*Fusarium*	*Botrytis*	6	6	6	na	SM production	Campbell et al., 2012, 2013
fHANT-AC for nitrate assimilation	Oomycetes	Dikarya	3	3^d^	3	2	Nutrient acquisition	Slot and Hibbett, 2007
fHANT-AC for nitrate assimilation	*Ustilago*	*Trichoderma*	3	3	3	2^e^	Nutrient acquisition	Slot and Hibbett, 2007
Fumonisin biosynthesis	*Fusarium*	*Aspergillus*	16	11	2	na	SM production	Khaldi and Wolfe, 2011
Fumonisin biosynthesis		Repeated transfer of 16–17-gene cluster between *Fusarium spp*.^f^					SM production	Proctor et al., 2013
*GAL* utilization	*Candida*	*Schizosaccharomyces*	5–6	3–4	4	4	Nutrient acquisition	Slot and Rokas, 2010
Gentisate catabolism	Between *Cochliobolus* and *Magnaporthe* grass pathogens^g^		6	6	6	na	Protection/defense	Greene et al., 2014
Gliotoxin and related ETP toxins	Multiple HGTs within Pezizomycotina		unknown^h^			18^i^	SM production	Patron et al., 2007^j^
Sterigmatocystin biosynthesis	*Aspergillus*	*Podospora*	23	24	23	23	SM production	Slot and Rokas, 2011
Tyrosine degredation	*Exophiala*	*Baudoinia*	5	8	4	na	Protection/defense	Greene et al., 2014

a *Donor and recipient taxonomic clade based on taxon sampling of each study*.

b *Number of MCG genes, where column D is the number of genes in the existing MGC in the donor lineage, R is the number of genes in the existing MGC in the recipient lineage, T is the number of gene trees supporting MGC-HGT reported by the original reference, and C is the number of gene trees supporting MGC-HGT confirmed by Richards et al. (2011)*.

c *See also Moore et al. (2014) which argues that extensive gene duplication and loss could also explain the ACE1 gene phylogenies*.

d *Genes are not clustered in some fungal lineages*.

e *Reported phylogenies for nitrate reductase and the high affinity nitrate transporter*.

f *Transfers inferred based on phylogenetic incongruence between accepted species phylogeny and supermatrix tree of concatenated genes in fumonisin MGC*.

g *Insufficient phylogenetic evidence to infer the direction of HGT event*.

h *Patron et al. (2007) lists two possible patterns of MGC inheritance: one via HGT and the other via vertical inheritance involving multiple gene duplications and losses*.

i *Richards et al. (2011) does not reject the hypothesis of HGT, but states that the extensive differences between the gene histories and the species phylogeny make it difficult to differentiate between HGT over complex gene loss*.

j *See also Ballester et al. (2014) which describes phylogenetic patterns indicative of HGT in the ETP MCG in Penicillium expansum and Penicillium roqueforti*.

To quantify this association between fungal MGCs and HGT, a recent survey of metabolic genes from 208 fungal genomes and HGT showed that genes in MGCs were transferred 1.66 fold more often than their non-clustered counterparts (Wisecaver et al., 2014), with clustered genes involved in SM biosynthesis showing significantly higher rates of HGT than other clustered genes in filamentous fungi. Taken together, these studies suggest that the organization of metabolic pathways into discrete MGCs may facilitate their dispersal through HGT.

## Ecological currency—adaptation may drive the gain and loss of metabolic gene clusters

By circumscribing the available niches in which fungal species and their MGCs interact and evolve, ecology is a major determinant of HGT of fungal MGCs, even though deciphering the ecological “means and motive” associated with specific HGT events is not always straightforward. This is largely due to the fact that HGTs are inferences of ancient historical events. For example, the exact MGC donors and recipients can never be known with certainty, unless the HGT event is extremely recent; thus, in most cases putative donor and recipient species are instead approximated based on extant sequenced genomes, and the likely ecological circumstances associated with the event are deduced from what is known about their organisms' ecologies. Recurrent HGT events, as may be the case for the gliotoxin and related ETP toxin MGCs (Patron et al., 2007), as well as very ancient HGT events render such interpretations particularly difficult. For example, the fHANT-AC cluster for nitrate assimilation may have first evolved in oomycetes, fungal-like microorganisms actually related to brown algae and diatoms, before it was transferred to the ancestor of Dikarya fungi, making the task of inferring the ancient selective pressures that may have driven this HGT event challenging (Slot and Hibbett, 2007). Nevertheless, the suggested age of the fHANT-AC transfer (circa 500 Ma) is consistent with the estimated age of the plant colonization of dry land (Sanderson et al., 2004), leading Slot and Hibbett (2007) to speculate that the ability to utilize nitrate as a source of fixed nitrogen may have been a key innovation that facilitated the Dikarya to radiate alongside plants across terrestrial habitats.

Limited functional understanding of MGC constituent genes also confounds ecological interpretation of HGT. This is particularly challenging for MGCs involved in secondary metabolism, because they are often lineage-specific and their enzymatic activities are often poorly characterized. Inferences can be made based on homology, but there are limitations. For example, Khaldi et al. (2008) suggests that the ACE1 cluster may have been horizontally transferred from *Magnaporthe* to *Aspergillus*. In the rice blast fungus *Magnaporthe grisea*, the ACE1 cluster is expressed during fungal penetration of host leaves, suggesting its involvement in plant pathogenicity (Böhnert et al., 2004); however, *Aspergillus clavatus* is not a plant pathogen, indicating the MGC likely serves a different function in this species.

Despite the challenges of identifying and interpreting past HGT, examination of well-characterized HGT events involving fungal MGCs shows that they occur in a wide variety of ecological settings and may involve species with overlapping ecological niches. Putative examples include HGT between ubiquitous fungal saprobes (Khaldi et al., 2008; Khaldi and Wolfe, 2011; Slot and Rokas, 2011), natural fermenters (Slot and Rokas, 2010), and plant pathogens (Campbell et al., 2012; Greene et al., 2014). In one instance, a fungal MGC encoding the tyrosine degradation pathway shows evidence of HGT from *Exophiala* to *Baudoinia*, both of which are thermotolerant, as well as between *Cochliobolus* and *Magnaporthe*, both of which are grass pathogens (Greene et al., 2014).

## Evolutionary outcomes—metabolic gene clusters follow different paths after transfer

**Figure 1:**
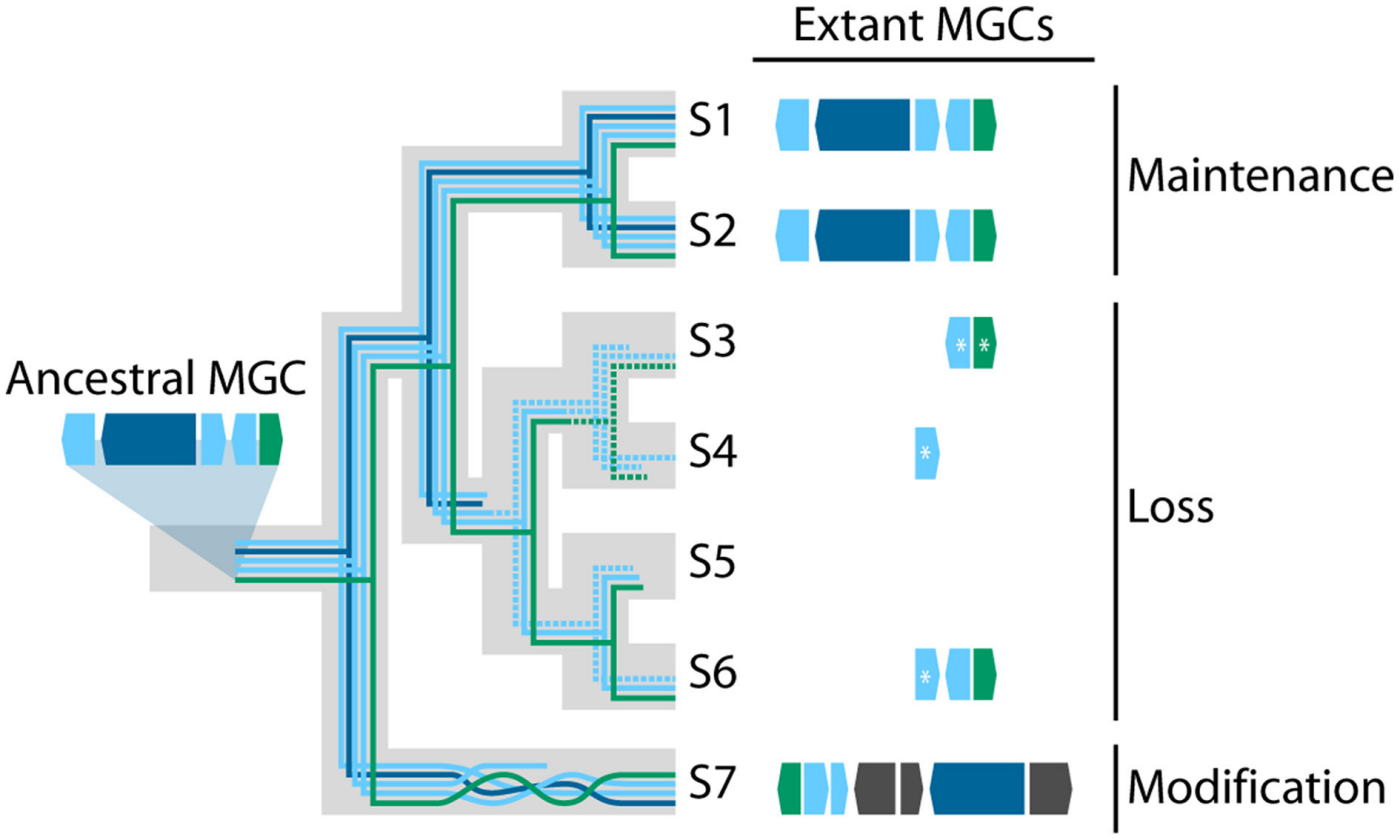
**Three evolutionary fates of transferred MGCs**. In this hypothetical example, a five gene MGC is transferred into the common ancestor of seven species of fungi (taxa S1–S7). The large gray tree represents the underlying species phylogeny, and each colored line represents the evolutionary history of a gene within the MGC. Dashed lines indicate genes that have been pseudogenized and are no longer functional. Extant MGCs in taxa S1 and S2 have maintained the same number of genes in the same orientation as the ancestral MGC suggesting purifying selection has acted to preserve the MGC's original form and function. In contrast, taxa S3–S6 contain the MGC in varying stages of decay, indicative of neutral evolution or positive selection for its loss. Asterisks (^*^) indicate pseudogenes. Finally, the MGC in taxon S7 has been modified from its original form, having undergone gene rearrangement, gene loss, and recruited three additional genes (colored in dark gray), which suggests the MGC has experienced diversifying selection.

**Figure 2:**
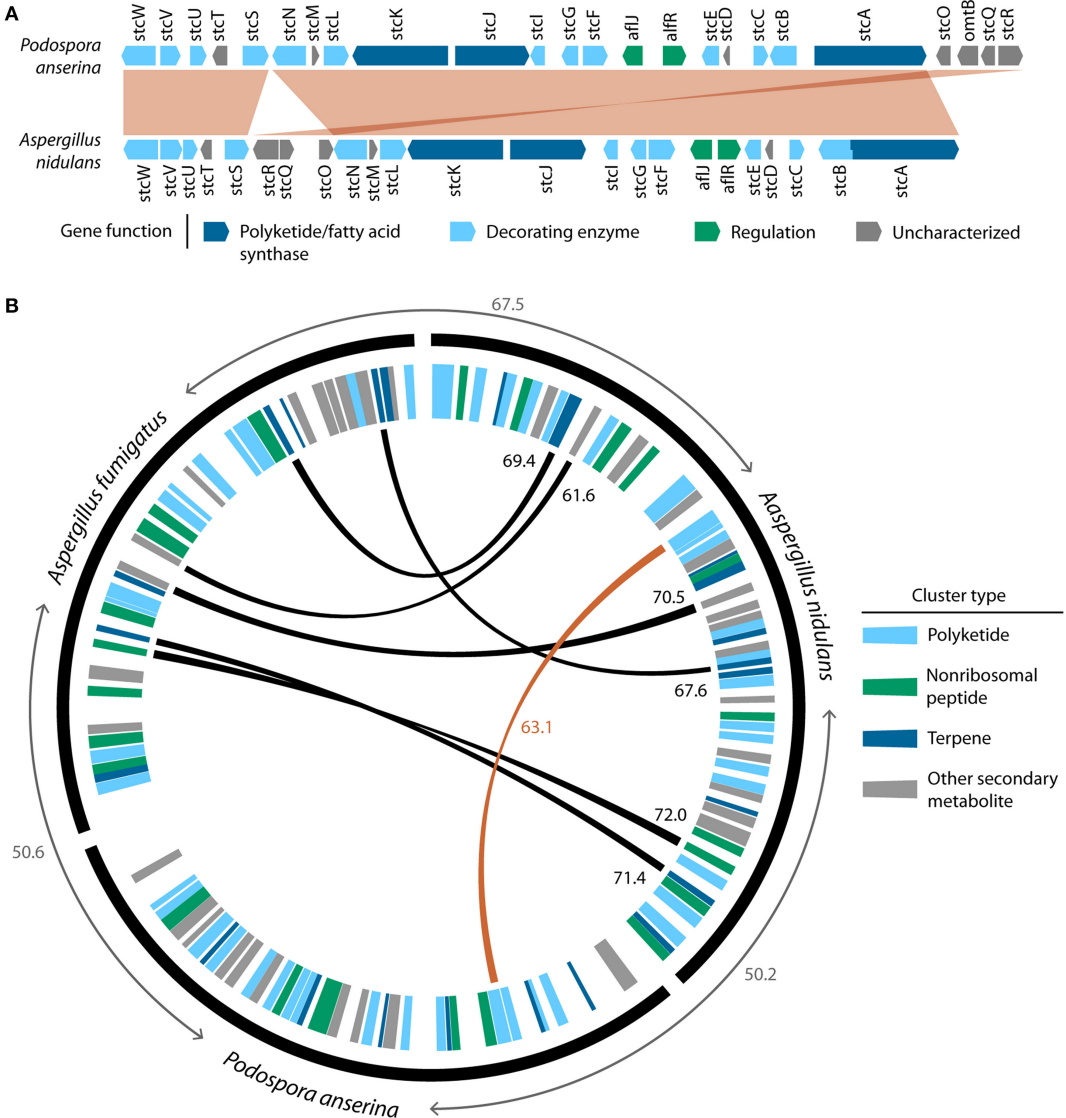
**The remarkable similarity of the sterigmatocystin MGC between *Aspergillus nidulans* and *Podospora anserina*, two organisms belonging to different fungal classes, is evidence of HGT. (A)** Synteny conservation between the sterigmatocystin MGC from *Podospora anserina* and *Aspergillus nidulans*. Aligned regions were drawn using the progressive Mauve algorithm (Darling et al., 2010) and are shown in red. **(B)** Conservation of SM gene clusters between *Podospora anserina, Aspergillus nidulans* and *Aspergillus fumigatus*. Circular tracks were created using Circos (http://circos.ca); the outer black track shows the relative gene counts in each of the three species. However, to visualize the relative location of SM gene clusters, the width of genes in these clusters have been drawn at 20 times the width of unclustered genes. Average amino acid percent identities (%IDs) of all reciprocal best BLAST hits (RBBHs) between the three genomes are shown in gray. SM gene clusters were predicted using antiSMASH (Blin et al., 2013), and SM cluster type is indicated by the colored wedges of the inner track. SM clusters were considered homologous if greater than 50% of their genes were RBBHs. Black links and black numbers indicate homologous SM clusters between *Aspergillus nidulans* and *Aspergillus fumigatus* and the average %IDs of RBBHs of the clustered genes, respectively. The red link and red number indicate the only homologous SM cluster (sterigmatocystin) identified between *Aspergillus nidulans* and *Podospora anserina* and the average %ID of RBBHs of the clustered genes, respectively. There are no homologous SM clusters between *Aspergillus fumigatus* and *Podospora anserina*.

Once integrated into a new genome, a horizontally transferred MGC may follow diverse evolutionary paths (Figure 1). In some cases, the original function of the MGC may be beneficial to the recipient organism, and purifying selection may act to preserve it. For instance, the MGC responsible for the biosynthesis of the SM sterigmatocystin, a highly carcinogenic mycotoxin, was likely horizontally transferred from *Aspergillus* to *Podospora* (Slot and Rokas, 2011). As the sterigmatocystin MGCs of *Podospora anserina* and *Aspergillus nidulans* are remarkably conserved in both synteny and sequence identity (Figure 2), one may infer strong selection for maintaining the MGC's structure and function in both the donor and the recipient. Similarly, strong purifying selection is the most likely explanation for the conservation of the nitrate reductase MGC across many fungal lineages for half a billion years (Slot and Hibbett, 2007).

The selection pressure may often differ across descendant lineages of the HGT recipient, and in some cases neutral evolution or even positive selection may result in partial or complete loss of a horizontally transferred MGC. For example, work by Campbell et al. (2012, 2013) suggests that the MGC for the biosynthesis of the SM bikaverin was horizontally transferred from *Fusarium* to *Botrytis*. Although a functional bikaverin MGC was identified in a rare *Botrytis cinerea* isolate (Schumacher et al., 2013), the MGC is more commonly present in various stages of decay in the genomes of several different *Botrytis* species (Campbell et al., 2013); for example, *Botrytis galanthina* and *Botrytis elliptica* retain only a few pseudogenes, whereas four other species appear to have lost the entire MGC altogether (Campbell et al., 2013).

Alternatively, once transferred into a new genome, fungal MGCs may experience diversifying selection that alters their functions. Although potentially the most interesting mode of selection, because of its ability to lead to the generation of novel pathways and metabolic products, diversifying selection is also the most challenging to identify and characterize because donor and recipient MGCs may appear very different following diversification. For example, Khaldi and Wolfe (2011) argued that the fumonisin MGC in *Aspergillus niger* was acquired via HGT from *Fusarium* but that the MGCs have since diverged in function, because the number and order of genes in the MGCs is no longer conserved. Similarly, diversifying selection following HGT may have also played a role in the discontinuous distribution of epipolythiodioxopiperazine (ETP) MGCs responsible for the fungal production of gliotoxin, sirodesmin and related mycotoxins. Patron et al. (2007) identified putative ETP gene clusters in diverse fungal species, but the MGCs varied dramatically in total gene length, suggesting they were responsible for the production of various SM products.

## Cellular trade routes—possible mechanisms for the acquisition of foreign DNA

Ecological opportunity and motive aside, identifying the possible cellular mechanisms for the uptake and incorporation of foreign DNA is equally important when seeking to understand the effect of HGT on fungal genomes. Recent reviews by Richards et al. (2011) and Soanes and Richards (2014) list several observable mechanisms for fungal HGT, including conjugation, both natural and agrobacterial mediated transformation, and viral transduction. Both reviews also cite data supporting conidial and hyphal fusion as potential fungal-specific routes for DNA exchange, arguing that, although vegetative incompatibility mechanisms would act to limit fusion events, errors in such systems need only occur at a low frequency to represent a viable mechanism for fungal HGT. Once taken up by the recipient cell, incorporation of foreign DNA could occur via ectopic repair of double-stranded DNA breaks, which genomes are susceptible to during cellular stress. Moreover, MGCs are often located in dynamic, rapidly evolving regions of fungal chromosomes, such as subtelomeres (Keller et al., 2005) or near mobile genetic elements (Han et al., 2001), both of which have been associated with instances of HGT in other eukaryotes (Gladyshev et al., 2008; Keeling and Palmer, 2008).

Another viable route of HGT for fungal MGCs is whole chromosome transfer. Several fungal species contain accessory chromosomes (also known as supernumerary or conditionally dispensable chromosomes), which are not essential for normal growth but may carry genes for specialized functions that are beneficial in certain conditions (Covert, 1998). An extreme example comes from the genome of the plant pathogenic fungus, *Mycosphaerella graminicola*, in which eight of the 21 chromosomes can be lost with no visible effect on the fungus and may have originated via HGT from an unknown donor (Goodwin et al., 2011). Another example comes from *Fusarium oxysporum* f. sp. *lycopersici*, which contains four accessory chromosomes that account for over 25% of its genome (Ma et al., 2010).

Importantly, accessory chromosomes have been shown to encode MGCs and can be transferred between fungal strains (He et al., 1998; Akagi et al., 2009; Ma et al., 2010; van der Does and Rep, 2012). The 1 Mb accessory chromosome in *Alternaria arborescens* contains 209 genes including ten putative MGCs (Hu et al., 2012). These 209 genes lack homology or have low sequence similarity to genes in other *Alternaria* spp. and have a different GC-content and codon bias compared to genes on essential chromosomes, leading Hu et al. (2012) to argue that the entire accessory chromosome, along with its ten MGCs, was obtained through HGT. Similarly, an accessory chromosome in some strains of *Nectria haematococca* contains a six gene pea pathogenicity (*PEP*) MGC required for causing disease on pea plants. Genes in the *PEP* cluster also have a different GC-content and codon bias compared to genes on essential chromosomes and may have been acquired via HGT (Temporini and Vanetten, 2004). Although this is an exciting and ongoing area of research in fungal biology, thus far horizontal chromosome transfer has only been documented between member of the same species or genus, so the relative impact of this process as a mechanism for fungal HGT is currently unknown.

## Evolutionist's toolkit—methods for detecting HGT

**Figure 3:**
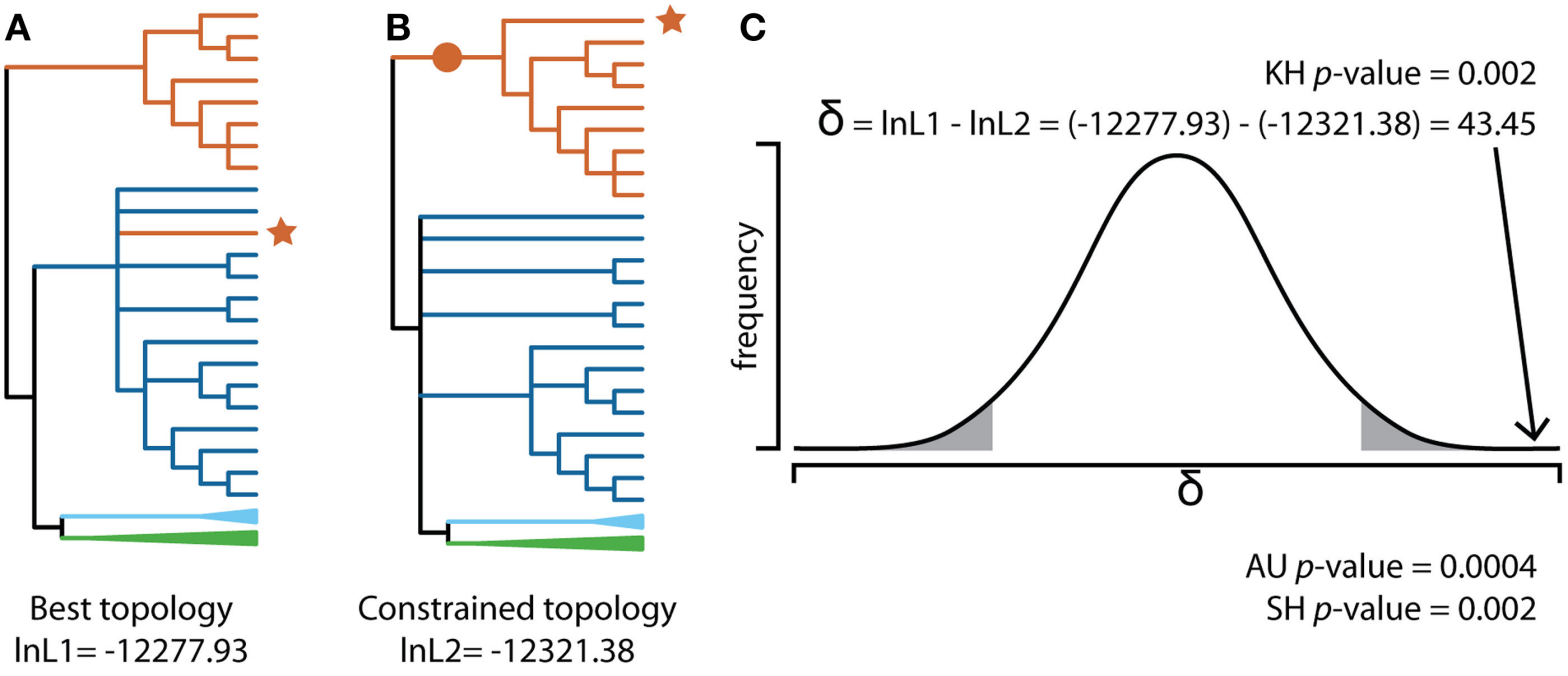
**Evaluating HGT using a comparative topology test. (A)** Maximum likelihood (ML) phylogeny of homologous sequences to stcI, a gene in the sterigmatocystin SM cluster, in *Podospora anserina* identified from a BLAST search of 161 Pezizomycotina genomes (JGI Mycocosm, download date 5 July 2014). Sequences were aligned and trimmed using MAFFT (Katoh and Standley, 2013) and trimAL (Capella-Gutierrez et al., 2009), respectively, and the phylogeny was created using RAxML (Stamatakis, 2014) using the PROTGAMMAAUTOF amino acid model of substitution and 100 bootstrap replicates. The resulting cladogram was midpoint rooted and branches supported by less than fifty bootstrap replicates were collapsed. This ML best tree depicts *Podospora anserina* (Sordariomycetes; red branch with red ^*^) grouping with Eurotiomycetes (dark blue branches). Other taxa in the phylogeny include additional Sordariomycetes (red branches), Leotiomycetes (light blue collapsed clade) and Dothideomycetes (green collapsed clade). **(B)** Best ML phylogeny using the same input data, but with the constraint imposed that *Podospora anserina* must group with other Sordariomycetes (red •). All other branches were resolved to obtain the maximum log-likelihood (-lnL), given the alignment using RAxML. **(C)** The difference in likelihood scores was evaluated to determine if the best topology represents a significantly better explanation of the data compared to the constraint topology. The Kishino–Hasegawa (KH) test (Kishino and Hasegawa, 1989) assumes a normal distribution of log-likelihood differences (δ). In this example, δ = 43.45 lies within the rejection region (gray area under curve) so one can reject the null hypothesis that the best topology is not statistically significantly better than the constraint topology (KH *p*-value = 0.002). Other tests, such as the Shimodaira–Hasegawa (SH) test (Shimodaira and Hasegawa, 1999) and the Approximately Unbiased (AU) test (Shimodaira, 2002), improved upon the KH test by correcting for multiple comparisons as well as the fact that the ML best tree is known *a priori*. In this example, both the SH test and AU test also reject the null hypothesis. All comparative topology tests were run in CONSEL (Shimodaira and Hasegawa, 2001).

Most cases of fungi-to-fungi HGT, including those involving MGCs, are first identified based on a sequence similarity search such as BLAST followed by a phylogenetic analysis, which demonstrates incongruence between gene trees and the established species phylogeny. Specifically, HGT is supported when genes suspected of being horizontally acquired have well-supported phylogenetic profiles that contradict accepted species relationships (Soanes and Richards, 2014). Whenever applicable, the hypothesis of HGT should be evaluated using a comparative topology test that examines whether the tree topology indicative of HGT is significantly better than other topologies that do not support the transfer event. Specifically, a comparative topology test compares the likelihood of the best topology to the likelihoods of one or more alternative topologies, given the sequence alignment, and computes the probability that the alternative topologies have a statistically lower fit to the data than the best topology (Figure 3, Shimodaira and Hasegawa, 2001). In the case of the sterigmatocystin MGC, comparative topology tests supported the placement of *Podospora* within or sister to the *Aspergillus* clade in nine out of the twenty three gene trees (Slot and Rokas, 2011).

Although many of the HGT events listed in Table 1 are supported by multiple phylogenetic and genomic comparisons, in some cases the available evidence does not allow for robust inference of HGT or its directionality. In some instances a MGC is shared between distantly related fungal species, indicating HGT likely played a part in its current distribution, but limited taxon sampling of closely related strains and species makes it difficult to resolve the timing and directionality of the HGT event (Patron et al., 2007; Greene et al., 2014). Many MGCs, particularly those involved in the production of SMs, have diversified rapidly (Carbone and Ramirez-Prado, 2007). As a consequence, some genes in MGCs share little or no sequence similarity to any other known sequence making it challenging to even construct gene trees with strong phylogenetic signals, let alone identify cases of HGT.

In contrast to MGC genes that are lineage-specific, other MGC genes are members of large, multi-copy, multi-domain gene families (Kroken et al., 2003; Bushley and Turgeon, 2010). Membership in such large, dynamically evolving gene families often makes distinguishing orthologs from paralogs extremely challenging (Haggerty et al., 2014). The complex evolutionary history of gliotoxin, a SM produced by *Aspergillus fumigatus* and other disparate fungal species, is a powerful illustration of this challenge. Patron et al. (2007) identified gliotoxin and gliotoxin-like MGCs in 14 species of Ascomycota. The resulting gene trees were extremely hard to reconcile with the species phylogeny, suggesting a complex evolutionary history likely due to a combination of gene duplication, transfer, and loss (Patron et al., 2007). A second phylogenetic analysis with increased taxonomic coverage by Richards et al. (2011) re-analyzed the evidence for HGT for this MGC, but was still unable to conclusively differentiate between HGT and complex patterns of gene loss. Phylogenetic patterns indicative of HGT were also found for genes in the gliotoxin-like MCGs of *Penicillium expansum* and *Penicillium roqueforti* (Ballester et al., 2014).

A perhaps more general approach to evaluate the evidence for HGT takes advantage of gene tree-species phylogeny reconciliation algorithms (Abby et al., 2010; Stolzer et al., 2012). For example, the phylogenetic software Notung can assign costs to HGT, gene duplication, and gene loss and use those costs to determine the most parsimonious combination of these three events to explain a given gene tree topology given the consensus species phylogeny (Chen et al., 2000; Vernot et al., 2007; Stolzer et al., 2012). In such analyses, HGT events will be inferred when a gene tree topology that is contradictory to the species phylogeny cannot be more parsimoniously reconciled using a combination of gene duplications and losses. This general approach is heavily dependent on the relative costs assigned to the three evolutionary events under consideration, namely HGT, gene duplication, and gene loss. Thus, it is important to evaluate how the relative costs assigned to the different events influence HGT inference. As an example, we reconciled the gene tree of homologous sequences to stcI (a gene in the sterigmatocystin SM cluster) in *Aspergillus* and *Podospora anserina* to the consensus Pezizomycotina species phylogeny using different costs for HGT. Only when using a HGT cost over 11.33 times the duplication cost, does Notung no longer predict a HGT from *Aspergillus* to *Podospora anserina* (Table 2).

**Table 2:** **Example gene tree-species phylogeny reconciliation analysis using Notung v2.8 (Vernot et al., 2007; Stolzer et al., 2012)**.

**Notung run**	**Transfer cost**	**Duplication cost**	**Loss cost**	**No. equally parsimonious solutions**	**Reconciliation score**	**Recovers *Podospora* HGT**
Figure S1C	-	1.5	1	1	123	no
Figure S1D	3	1.5	1	256	70	yes
Figure S1E	7.5	1.5	1	1	97.5	yes
Figure S1F	15	1.5	1	1	112.5	yes
Figure S1G	17	1.5	1	1	116.5	yes
Figure S1H	19	1.5	1	1	119.5	no
Figure S1I	30	1.5	1	1	123	no

*Gene tree of homologous sequences to stcI (a gene in the sterigmatocystin SM cluster, Supplementary Figure S1A) was reconciled to the consensus species phylogeny (Supplementary Figure S1B) using different transfer costs. Only when using a transfer cost over 11.33 times the duplication cost, does Notung no longer predict a HGT from Aspergillus to Podospora anserina. See Supplementary Figure 1 for reconciled trees*.

Because the phylogenetic signal in single gene phylogenies can be weak (Rokas et al., 2003) and a wide variety of other biological and methodological factors can also produce gene trees that differ from the species phylogeny (Salichos and Rokas, 2013), examination of the concordance of evolutionary histories of genes in MGCs and their joint difference from the species phylogeny can significantly strengthen inference of HGT, something that is obviously impossible to do in examinations of single gene HGT events. For example, further support for the HGT of the sterigmatocystin MGC comes from a pairwise comparison of the clusters in *Podospora anserina* and *Aspergillus nidulans*, which shows an extreme conservation of gene order as well as increased sequence similarity of the sterigmatocystin gene orthologs compared to the average sequence similarity of all gene orthologs (Figure 2, Slot and Rokas, 2011). Taken together, the topology tests, gene tree-species phylogeny reconciliation, and unusually high conservation in gene content, sequence, and microsynteny, all provide strong support for the HGT of this gene cluster. Moreover, Slot and Rokas (2011) also demonstrated conservation of a cis-regulating element between *Podospora anserina* and *Aspergillus nidulans* as well as evidence of expression of the sterigmatocystin MGC in *Podospora anserina*, which coupled with the fact that *Podospora* produces sterigmatocystin (Matasyoh et al., 2011), suggests that HGT resulted in the acquisition of a functional cluster in this species.

Detection of a rare genomic change shared between donor and recipient taxa can provide additional evidence for a putative HGT event (Moran and Jarvik, 2010; Li et al., 2014). In the case of the horizontally acquired *GAL* cluster in *Schizosaccharomyces*, the *GAL10* gene contains fused epimerase and mutarotase domains, a trait shared with putative MGC donors but absent in other fungal species (Slot and Rokas, 2010). Remarkably, *Schizosaccharomyces* species still retain a vertically inherited, unclustered *GAL10* paralog as well that contains only an unfused epimerase domain (Slot and Rokas, 2010).

## No more analogies—conclusions and perspective

Studies suggest that several fungal MGCs have been transferred and have followed diverse evolutionary paths across a wide range of fungi (Table 1) that vary in their ecological life history strategies, including opportunistic animal and plant pathogens, suggesting that HGT of MGCs is a source of gene innovation in fungal specialized metabolism. Given that most fungal genomes have yet to be fully explored, that many fungal MGCs—especially those that produce SMs—exhibit very narrow taxonomic distributions, and that circumscription of novel MGCs continues unabated, it seems likely that the current number of known cases of HGT of fungal MGCs is just the tip of the iceberg.

Although it is to be expected that many more cases of HGT involving MGCs are going to be discovered in the years to come, determining the rate of such events as well as the evolutionary fates of the transferred MGCs remains an ambitious goal. What is sorely needed are targeted, in-depth sequencing efforts of closely related strains and species that might capture the remarkably rapid turn-around of fungal MGCs, coupled with functional studies and improved algorithms that characterize the number and function of MGCs in those genomes.

The small number of reported HGT cases, most of which are very ancient, severely limits efforts to understand the ecological circumstances that underlie these exchanges and how they alter the structure of fungal communities. At present, we still lack any understanding as to whether particular ecological lifestyles are more prone to HGT events than others. *A priori*, one might expect that fungi that form intimate ecological associations such as mycoparasites, endophytes and lichens might be more prone to HGT than free-living fungi, but the relative dearth of genome representatives for most fungal lifestyles severely limits the scope of any such analysis.

Although several mechanisms for both the acquisition and assimilation of foreign DNA exist in fungi (Ma et al., 2010; Soanes and Richards, 2014), the relative contribution of these mechanisms to the observed patterns of HGT is unclear; moreover, the mechanisms for stages of HGT between cell entry and genome incorporation are completely uncharacterized (Scazzocchio, 2014). For example, how and how frequently does foreign DNA escape nucleases and how does foreign DNA enter the nucleus? Is the chance of successful integration associated with foreign DNA length and is there bias toward integration of smaller fragments? At what rate is foreign DNA acquired, and how does this rate vary across species and environments? Once integrated, what fraction of transfers have a measurable fitness effect, and what fraction of those are beneficial vs. deleterious? Do horizontally acquired MGCs become integrated into existing regulatory networks of metabolism or does the acquisition of MGCs precipitate the modification of existing metabolic and regulatory networks?

These outstanding questions on the prevalence and consequences of HGT of fungal MGCs, as well as the ecology and mechanisms driving transfer events, require systematic tests of hypotheses about the process that often require integrative data from diverse facets of fungal biology, from genomics to chemistry, and from ecological lifestyles to evolutionary history. Fortunately, the ever larger number of fungal genomes sequenced and the increasing importance given to the sampling of ecologically and taxonomically important groups, coupled with novel functional genomic technologies, is quickly bringing remarkable amounts of data to bear on these questions. For example, the 1000 fungal genome project (http://1000.fungalgenomes.org), an ongoing community sequencing initiative sponsored by the Joint Genome Institute, aims to sequence at least two genomes from each of the more than 500 fungal families, including genomes representative of all the major fungal lifestyles and morphologies; similarly, the yeast 1000 project, recently funded by the National Science Foundation (http://www.nsf.gov/awardsearch/showAward?AWD_ID=1442148), aims to decode the genomes of all ~1000 yeast species belonging to the subphylum Saccharomycotina. Such projects promise to not only serve as comprehensive surveys of fungal metabolism but also provide unprecedented opportunities for evaluating and quantifying the impact that processes, such as HGT-mediated gene innovation, have had on the generation of fungal biodiversity.

### Conflict of interest statement

The authors declare that the research was conducted in the absence of any commercial or financial relationships that could be construed as a potential conflict of interest.
