# Supplementary material for: Fungal metabolic gene clusters—caravans traveling across genomes and environments
Source: Front Microbiol. 2015 Mar 3;6:161. doi: 10.3389/fmicb.2015.00161 (PMC4347624; doi:10.3389/fmicb.2015.00161)
Supplement: Supplementary file 1 [file Image1.PDF]

Supplementary Material

Fungal metabolic gene clusters – caravans traveling across genomes and environments

Jennifer H. Wisecaver and Antonis Rokas*

Department of Biological Sciences, Vanderbilt University, Nashville, TN, USA

*** Correspondence:** Antonis Rokas, Department of Biological Sciences, Vanderbilt University, VU Station B 351364, Nashville, TN 37235, USA, antonis.rokas@vanderbilt.edu

Supplementary Figure 1. Gene tree-species phylogeny reconciliation performed in Notung v2.8 (http://lampetra.compbio.cs.cmu.edu/Notung/index27.html). In all trees Sordariomycetes are indicated by the red taxa names, Eurotiomycetes by the dark blue names, Leotiomycetes by the light blue names, and Dothideomycetes by the green names.

(A) Consensus maximum likelihood (ML) Pezizomycotina species phylogeny based on the concatenated alignments of DNA-directed RNA polymerase II subunits RPB1 and RPB2 and elongation factor 1-alpha from 162 fungal genomes (JGI Mycocosm, download date 5 July 2014). Sequences were aligned and trimmed using MAFFT (Katoh and Standley, 2013) and trimAL (Capella-Gutierrez et al., 2009), respectively, and the phylogeny was created using RAxML (Stamatakis, 2014) using the PROTGAMMAAUTOF amino acid model of substitution and 100 bootstrap replicates. The resulting cladogram was rooted using *Saccharomyces cerevisiae* S288c as the outgroup and branches supported by less than 80 bootstrap replicates were collapsed.

(B) ML gene tree of homologous sequences to stcI, a gene in the sterigmatocystin SM cluster, in *P. anserina* identified from a BLAST search of 161 Pezizomycotina genomes (JGI Mycocosm, download date 5 July 2014). Sequences were aligned and trimmed using MAFFT (Katoh and Standley, 2013) and trimAL (Capella-Gutierrez et al., 2009), respectively, and the phylogeny was created using RAxML (Stamatakis, 2014) using the PROTGAMMAAUTOF amino acid model of substitution. Tree is midpoint rooted. This ML best tree depicts *P. anserina* (Podan23463_5145_Podan2) grouping within Eurotiomycetes.

(C) stcI gene tree reconciled to the consensus species phylogeny using a duplication cost of 1.5, loss cost of 1 and ILS cost of 0. The resulting reconciled phylogeny had a reconciliation score of 123, 18 duplication events (red internal nodes labeled D), and 96 losses (light grey branches).

(D) stcI gene tree reconciled to the consensus species phylogeny using a duplication cost of 1.5, transfer cost of 3, loss cost of 1 and ILS cost of 0. The resulting reconciled phylogeny had a reconciliation score of 70, 15 transfer events (yellow branches), 2 duplication events (red internal nodes labeled D), and 96 losses (light grey branches). Only showing 1 of 256 equally parsimonious solutions.

(E) stcI gene tree reconciled to the consensus species phylogeny using a duplication cost of 1.5, transfer cost of 7.5, loss cost of 1 and ILS cost of 0. The resulting reconciled phylogeny had a reconciliation score of 97.5, 2 transfer events (yellow branches), 11 duplication events (red internal nodes labeled D), and 66 losses (light grey branches).

(F) stcI gene tree reconciled to the consensus species phylogeny using a duplication cost of 1.5, transfer cost of 15, loss cost of 1 and ILS cost of 0. The resulting reconciled phylogeny had a reconciliation score of 112.5, 2 transfer events (yellow branches), 11 duplication events (red internal nodes labeled D), and 66 losses (light grey branches).

(G) stcI gene tree reconciled to the consensus species phylogeny using a duplication cost of 1.5, transfer cost of 17, loss cost of 1 and ILS cost of 0. The resulting reconciled phylogeny had a reconciliation score of 116.5, 2 transfer events (yellow branches), 11 duplication events (red internal nodes labeled D), and 66 losses (light grey branches).

(H) stcI gene tree reconciled to the consensus species phylogeny using a duplication cost of 1.5, transfer cost of 19, loss cost of 1 and ILS cost of 0. The resulting reconciled phylogeny had a reconciliation score of 119.5, 1 transfer events (yellow branches), 13 duplication events (red internal nodes labeled D), and 81 losses (light grey branches).

(I) stcI gene tree reconciled to the consensus species phylogeny using a duplication cost of 1.5, transfer cost of 30, loss cost of 1 and ILS cost of 0. The resulting reconciled phylogeny had a reconciliation score of 123, 0 transfer events (yellow branches), 18 duplication events (red internal nodes labeled D), and 96 losses (light grey branches).

References

Capella-Gutierrez, S., Silla-Martinez, J. M., and Gabaldon, T. (2009). trimAl: a tool for automated alignment trimming in large-scale phylogenetic analyses. *Bioinformatics* 25, 1972–1973. doi:10.1093/bioinformatics/btp348.

Katoh, K., and Standley, D. M. (2013). MAFFT Multiple Sequence Alignment Software Version 7: Improvements in Performance and Usability. *Mol. Biol. Evol.* 30, 772–780. doi:10.1093/molbev/mst010.

Stamatakis, A. (2014). RAxML version 8: a tool for phylogenetic analysis and post-analysis of large phylogenies. *Bioinformatics* 30, 1312–1313. doi:10.1093/bioinformatics/btu033.

Figure S1A

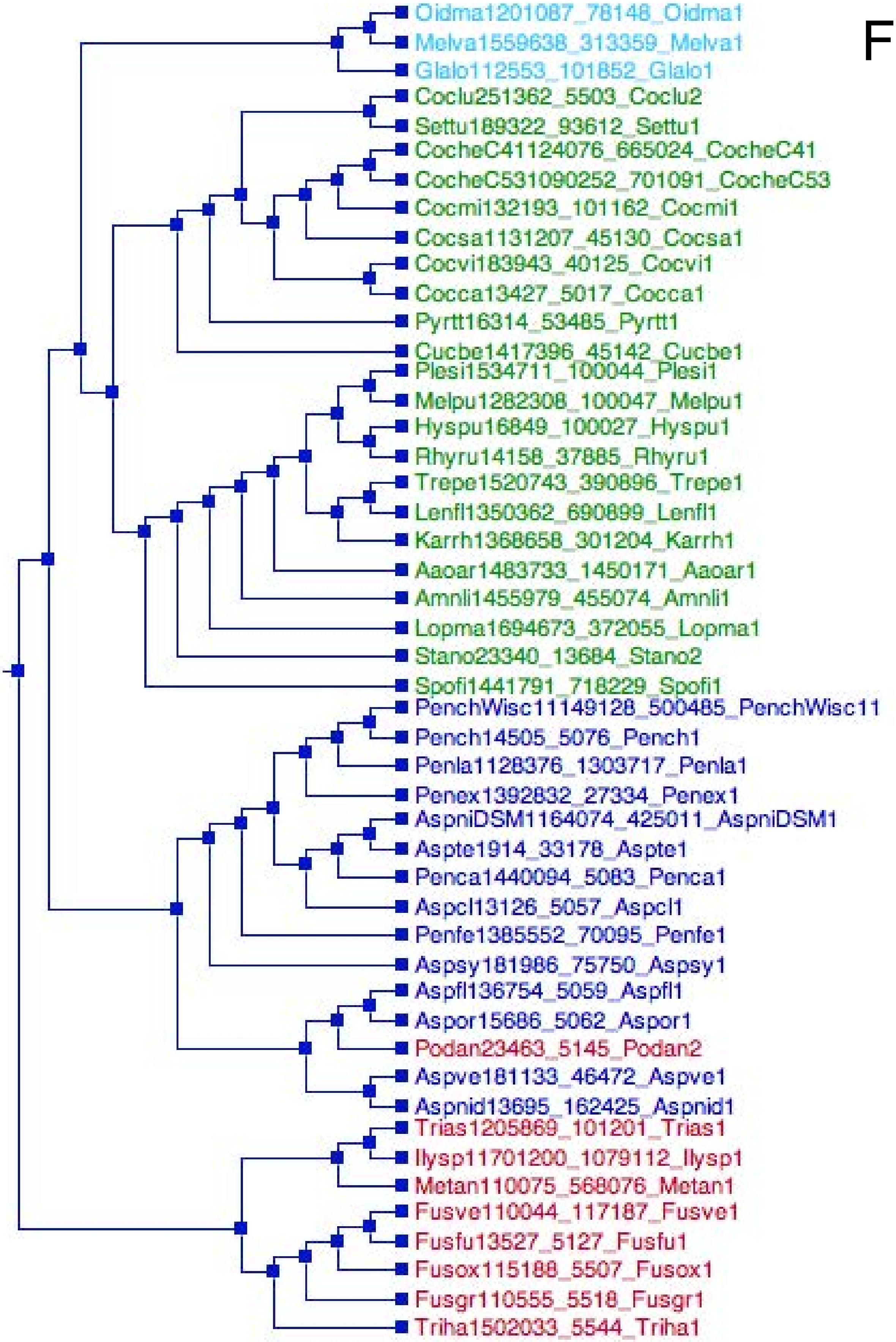

Image generated with Notung 2.8.1.2, on Jan 11, 2015

Figure S1B

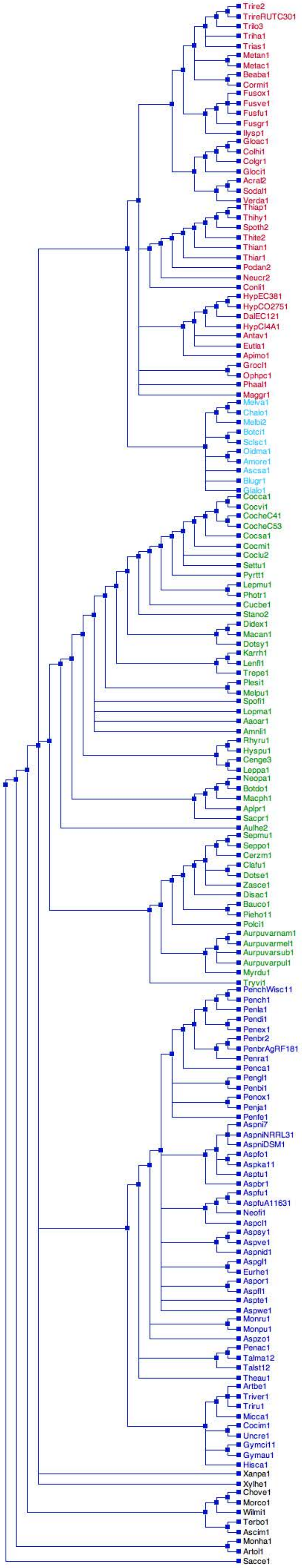

Image generated with Notung 2.8.1.2, on Jan 11, 2015

Figure S1C

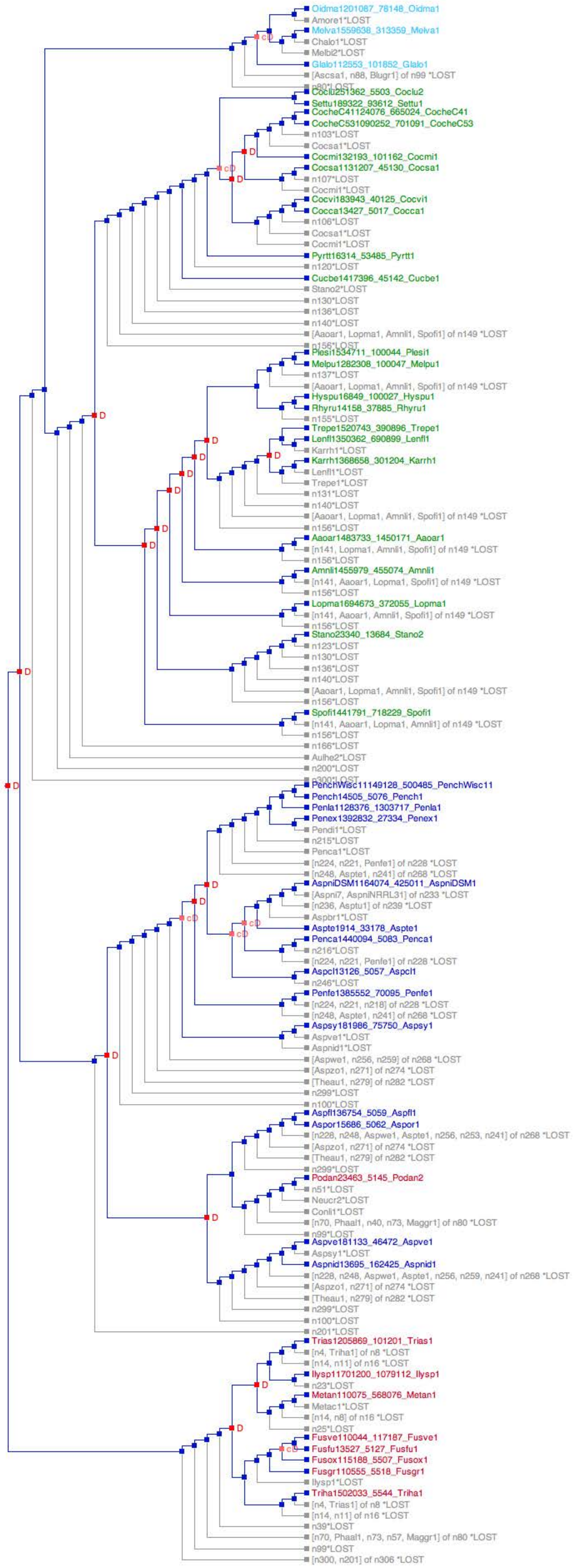

Image generated with Notung 2.8.1.2, on Jan 11, 2015

Figure S1D

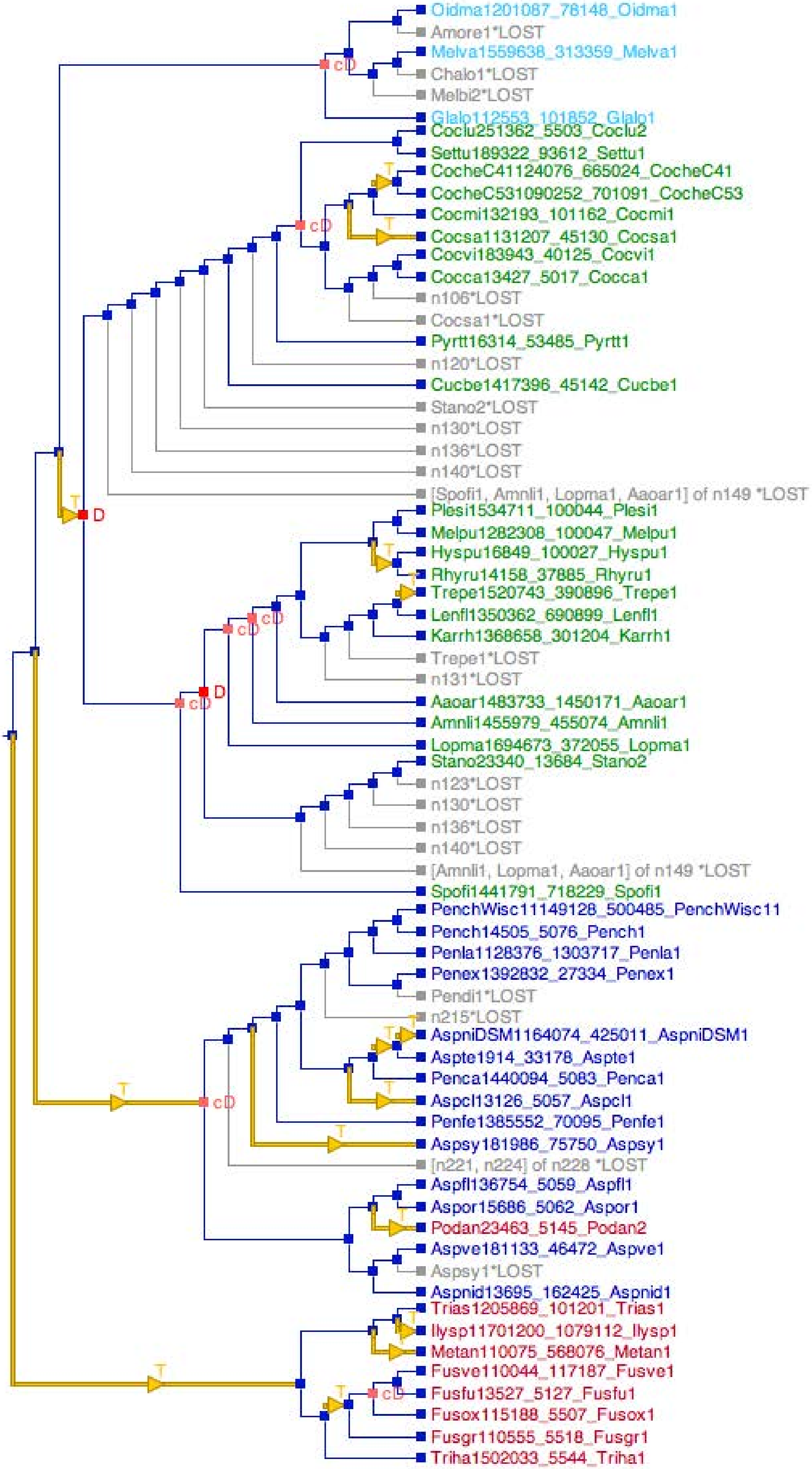

Image generated with Notung 2.8.1.2, on Jan 11, 2015

Figure S1E

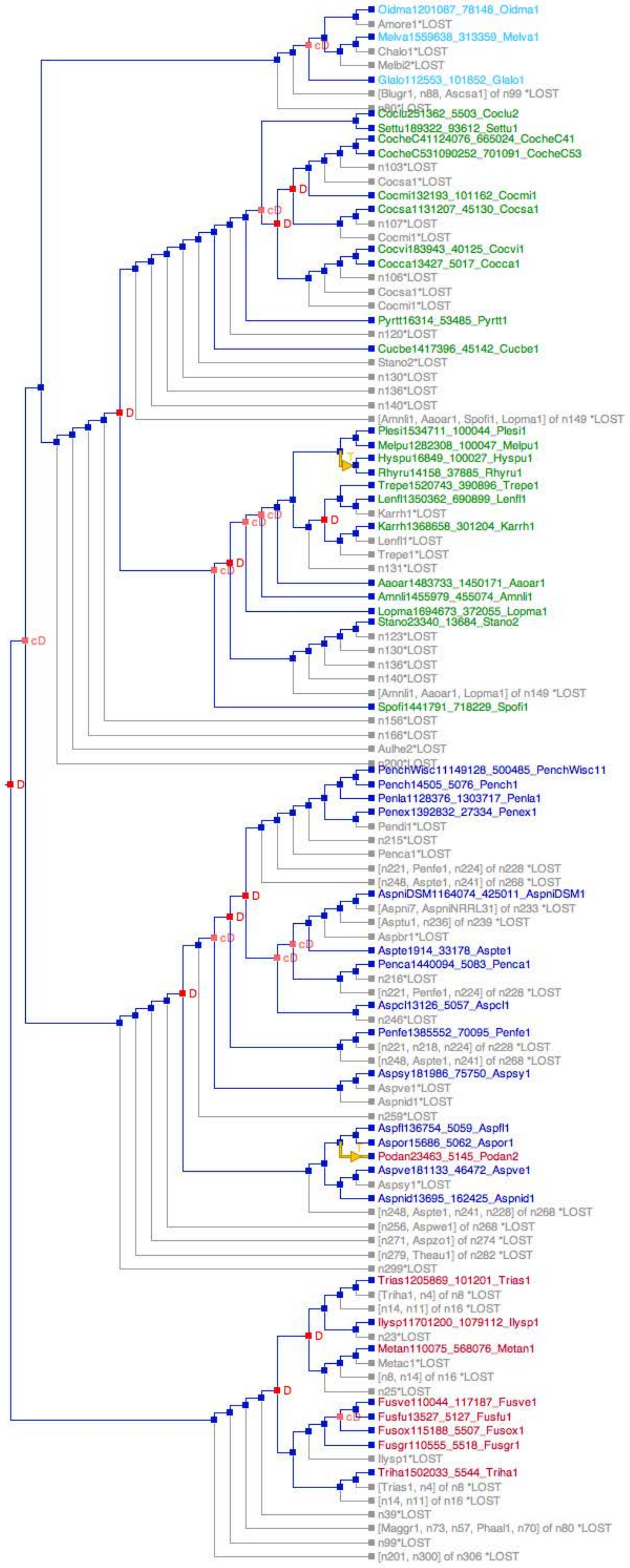

Image generated with Notung 2.8.1.2, on Jan 11, 2015

Figure S1F

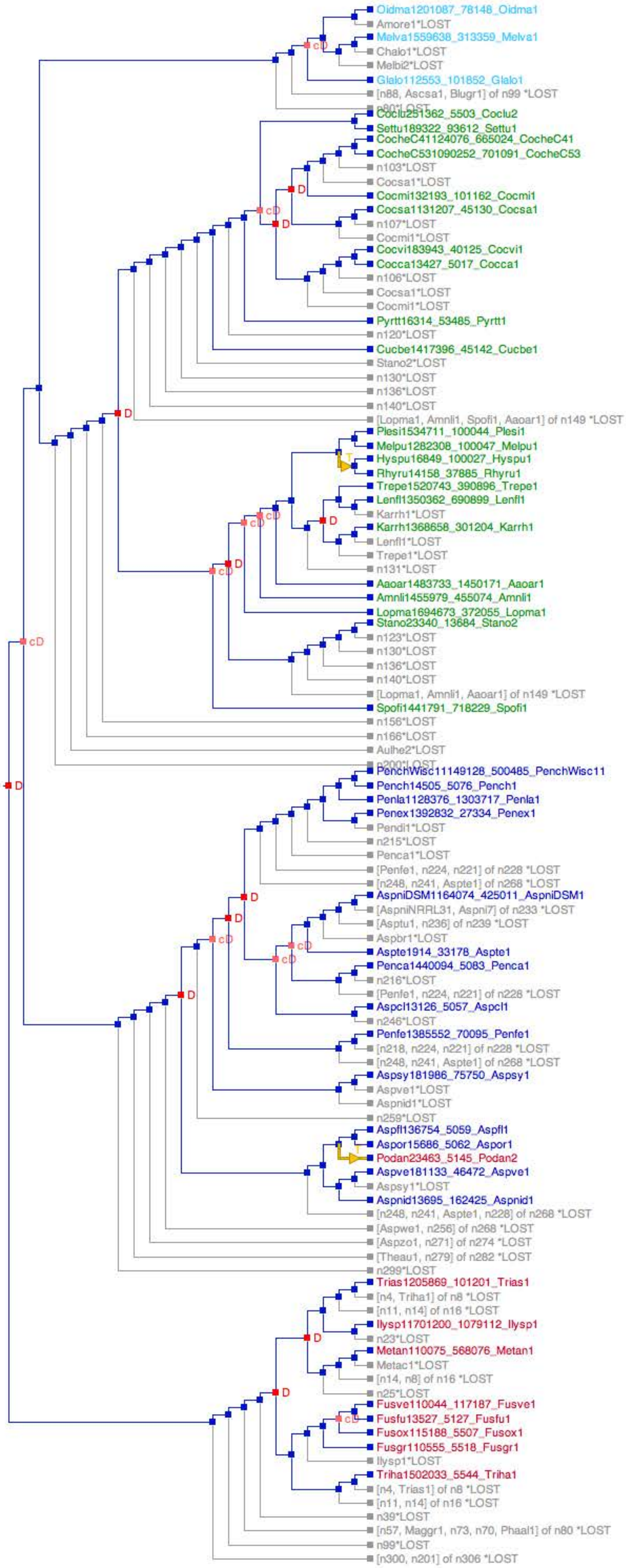

Image generated with Notung 2.8.1.2, on Jan 11, 2015

Figure S1G

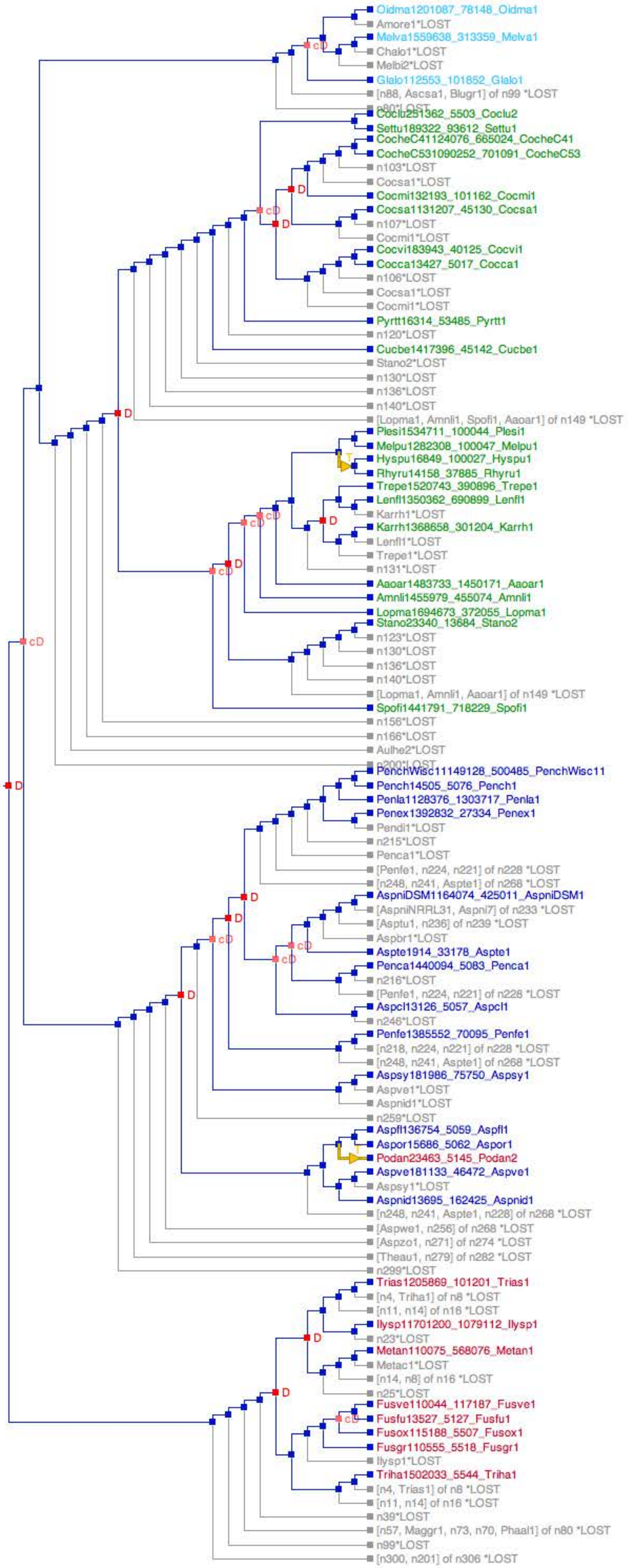

Image generated with Notung 2.8.1.2, on Jan 11, 2015

Figure S1H

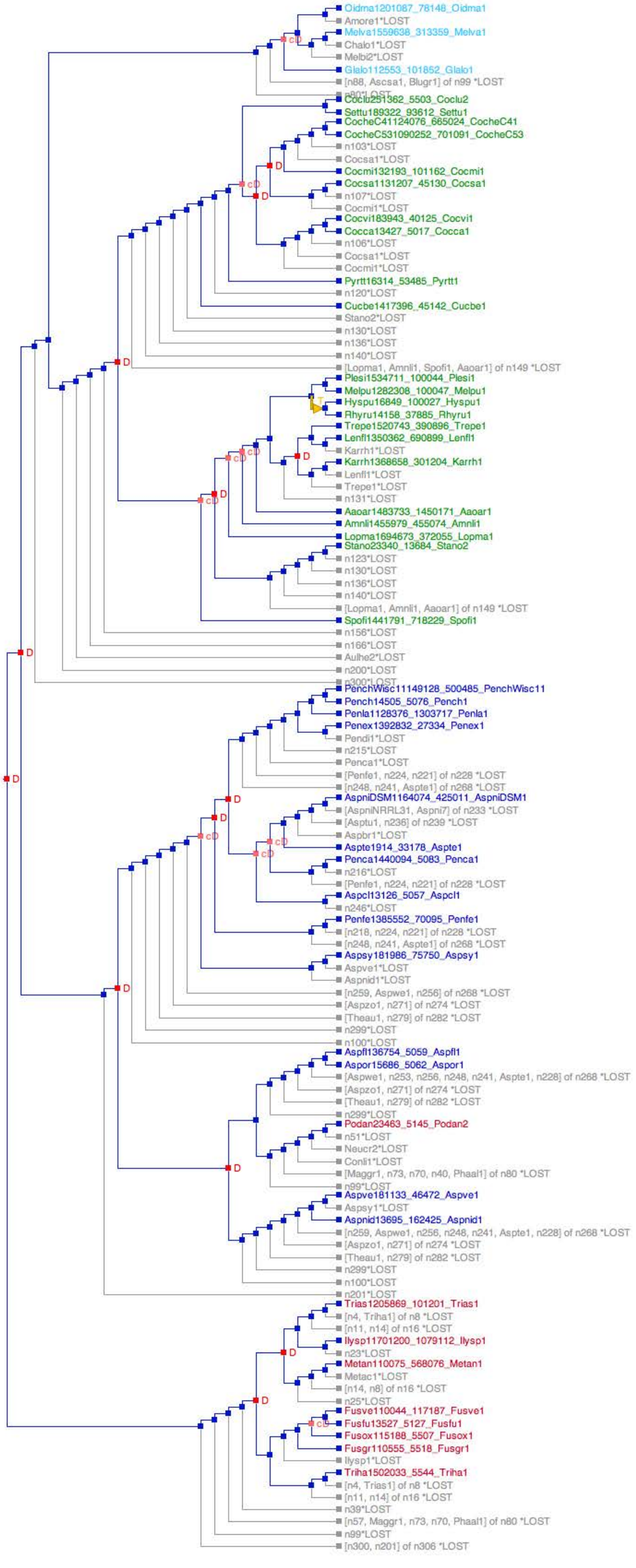

Image generated with Notung 2.8.1.2, on Jan 11, 2015

Figure S1I

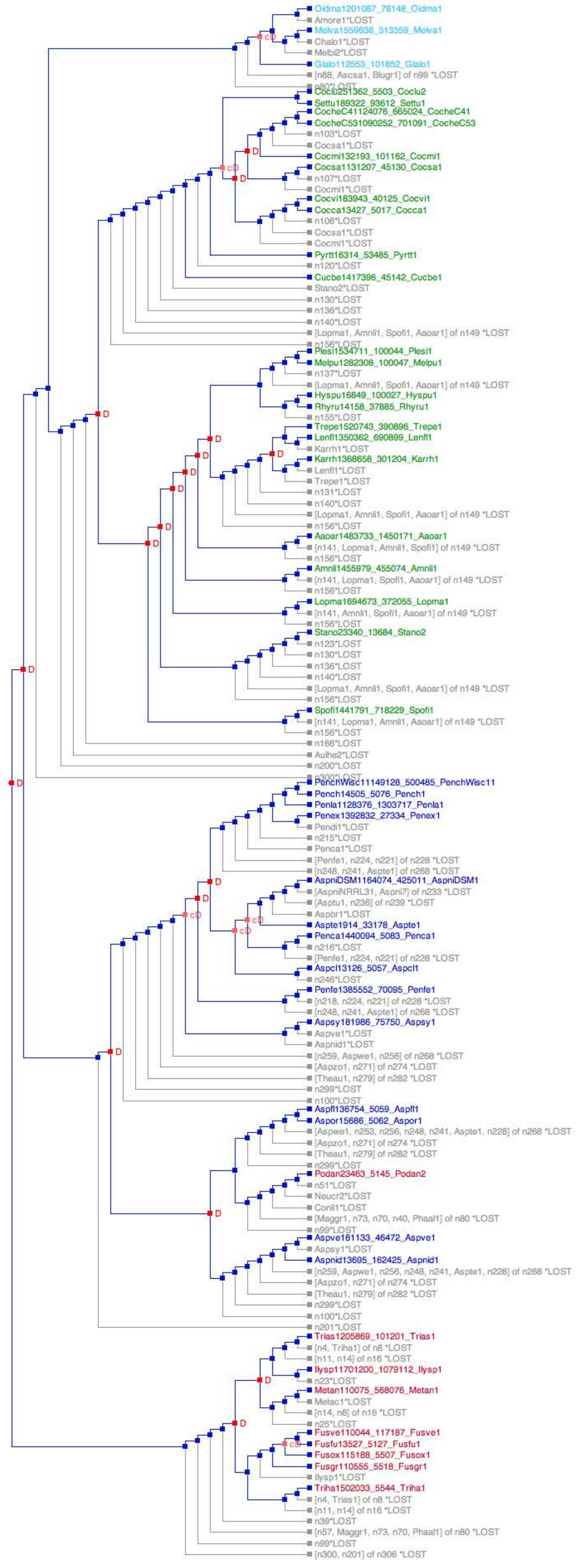

Image generated with Notung 2.8.1.2, on Jan 11, 2015
